# Investigation of the qualitative and appearance characteristics of *Eryngium caeruleum* L. based on colorimetric and browning indices in storage conditions

**DOI:** 10.1002/fsn3.4243

**Published:** 2024-07-02

**Authors:** Soudabeh Nourzad, Hassanali Naghdi Badi, Sepideh Kalateh Jari, Ali Mehrafarin, Sakineh Saeidi‐Sar

**Affiliations:** ^1^ Department of Horticultural Science and Agronomy, Science and Research Branch Islamic Azad University Tehran Iran; ^2^ Department of Agronomy and Plant Breeding, Faculty of Agriculture Shahed University Tehran Iran; ^3^ Medicinal Plants Research Center Shahed University Tehran Iran; ^4^ Department of Agricultural Science Technical and Vocational University (TVU) Tehran Iran

**Keywords:** ascorbic acid, drying methods, hue, phenolic compounds, reddish, sea holly

## Abstract

The eryngo plant is an herb related to the Apiaceae family with the greatest diversity of species, has a gorgeous taste when eaten as a vegetable, and is traditionally used in folk medicine for its health benefits. The present study was to assess the effects of different drying methods and storage times on the quality and appearance of *Eryngium caeruleum*. The treatments of this study were drying methods (room temperature (25°C) with proper ventilation, oven temperature 55°C, vacuum oven temperature 55°C, and a microwave with a power of 500 W) and storage times (1, 75, and 150 days). The lowest brightness index and the highest browning index were found in the shade‐dried samples kept in the refrigerator for 150 days. In these samples, the numerical values of chlorophyll were lower than others. After 75 days, in the vacuum oven‐dried samples, the maximum levels of total phenolics and flavonoids and antioxidant activity were calculated. The colorimetric test showed that the oven‐dried samples maintained their green color well. However, the storage decreased the quality of plant samples due to the degradation of chlorophyll. Overall, this study showed that the highest greenness and appearance qualities were found in the samples dried in a vacuum oven at 55°C. It seems that appearance indices can be introduced as an initial and quick step in the qualitative assessment of dried leafy products.

## Graphical abstract

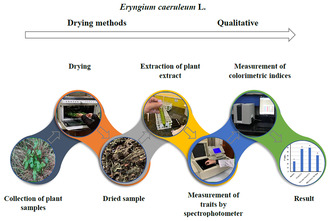

## 1 INTRODUCTION

Today, medicinal herbs occupy an important part of the food and pharmaceutical industry, which are rapidly expanding due to their simultaneous nutritional and medicinal value (Ivanišová et al., 2021). *Eryngium caeruleum* is one of the plants in the fields of natural resources that have received less attention despite the importance they can have in providing human health. Most *E. caeruleum* L. species, known for their synthesis of secondary polyphenols, are considered anti‐inflammatory, tonic, and antioxidant (Ghajarieh Sepanlou et al., 2019). *E. caeruleum* L. is an herbaceous and perennial plant, found abundantly in the humid and forested areas of northern Iran. It has long been used in folk medicine for its diuretic, expectorant, antiscorbutic, anti‐inflammatory, diaphoretic, and aphrodisiac effects, which can be used to treat anemia, gastrointestinal infections, and kidney stones (Keykhaee et al., 2022). The shelf life of these products can be severely reduced due to their high rate of respiration and transpiration, as well as their microbial and enzymatic activities (El‐Beltagi et al., 2023).

The quantity and quality of bioactive components depend on the genotype, habitat conditions, geographical origin, harvest time, organ, storage conditions, processing method, extraction method, and sometimes the type of solvent (Rossi et al., 2016). Drying is a common method for preserving food, vegetables, and medicinal plants. One of the most important advantages of drying plant materials, in addition to preventing chemical waste, is reducing the cost of storage and transportation (Alonge & Iroemeha, 2013). Changes in product quality during drying and storage depend on the type of drying operation and the accuracy of material preparation before drying, which is divided into four physical, chemical, microbial, and nutrient groups: (I) Physical changes: These include structural changes of the product, hardening of the shell, disintegration, and formation of holes, cracks, dehydration, and stickiness. (II) Chemical changes: These include browning, oxidation of fats, loss of color, and changes in the taste of food. (III) Microbial changes: the activity of microorganisms is very limited due to low water activity. (IV) Nutritional changes: the quality of nutrients can be affected during drying (Sablani et al., 2006).

Sensory characteristics of dried materials are also important in determining the quality of dried products, which include color, aroma, taste, texture, and taste. The aroma and taste can change due to the loss of volatile organic compounds, which is the most common form of quality reduction in this aspect. Low‐temperature drying is used for products with a high economic value, such as medicinal plants and spices (Bhatta et al., 2020). Low‐temperature dryers have been found to cause the least damage to leafy vegetables and retain more nutrients than other dryers. Furthermore, drying in the absence of oxygen preserves compounds sensitive to oxidation (Singh & Pandey, 2011). Considering that few studies have been carried out in the field of identification and exploitation of *E. caeruleum*, the aim of this research was to evaluate different drying methods on the appearance and quality of the aerial parts of this plant at different times of storage.

## 2 MATERIALS AND METHODS

This research was conducted in the spring of 2020 in order to evaluate the appearance quality of *E. caeruleum* samples, which were the re‐grown vegetative bodies of perennial specimens in Noor, Mazandaran, Iran. The experiment was conducted as a factorial with a completely randomized design with 3 replications. The first factor was different drying methods (in shade inside a room with a temperature of about 25 ± 3°C and proper ventilation, a 55°C oven (Memert ULM‐700), a 55°C vacuum oven (Memert, model, Vo‐600)), and a 500 W microwave (LG model MP‐9484WR), and the second factor was the various storage durations (1, 75, and 150 days). The traits studied were as follows. The diameter of the plant for drying was 2–5 cm. During the drying period, when the weight of the sample was fixed after weighing 3 times, the drying operation was stopped.

### 2.1 Chlorophyll content

To compare the content of chlorophylls, 0.5 g of plant material was crushed in 20 mL of 80% acetone, and then it was placed in a centrifuge at 6000 rpm for 10 min. The absorbance value was read separately at wavelengths of 663 nm for chlorophyll a, 645 nm for chlorophyll b, and 470 nm for carotenoids, then using the following formulas (Arnon, 1967).

(1) $$\text{Chlorophyll}\;a=\frac{\left(19.3\times A663-0.86\times A645\right)\;V}{100W}$$

(2) $$\text{Chlorophyll}\;b=\frac{\left(19.3\times A645-3.6\times A663\right)\;V}{100W}$$

(3) $$\text{Carotenoids}=\frac{100\left(A470\right)-3.27\left(\mathrm{mg}\;\mathrm{chl}.a\right)-104\left(\mathrm{mg}\;\mathrm{chl}.b\right)}{227}$$

where *V* is the volume of the filtered solution, *A* is the absorption of light at wavelengths, and *W* is the fresh weight of the sample.

### 2.2 Total phenolic and flavonoid content

For total phenolic, according to the method of McDonald et al. (2001), 0.5 mL of extract was mixed well with 5 mL of Folin‐Ciocalto reagent and 4 mL of one molar sodium carbonate solution, and the absorption was at a wavelength of 765 nm. The aluminum chloride colorimetric method was used to determine the content of flavonoids. Each of the plant methanolic extracts (half a milliliter of 1:10 g/mL) was separately mixed with 1.5 mL of methanol, 0.1 mL of aluminum chloride (10% methanol), 0.1 mL of potassium acetate (1 M), and 2.8 mL of distilled water. The absorbance of each reactive compound was measured at 415 nm (Chang et al., 2002).

### 2.3 Antioxidant activity

First, plant extracts at different concentrations of 5 × 10^2^–5 × 10^6^ mg/100 in pure methanol and a 1:1 mixture of DPPH (2,2‐Diphenyl‐Picryl‐Hydrazyl) solution (8 mg/100) and plant extracts with different concentrations were prepared. The absorbance of the samples was then measured at 517 nm after 30 m. The DPPH free radical inhibition percentage of the samples was obtained using the following equation (Sun et al., 2007).

(4) $$R\%=\frac{\mathrm{AD}–\mathrm{AS}}{\mathrm{AD}}\times 100$$

*R*%: percent inhibition; AD: absorbance of DPPH at 517 nm; AS: absorbance of samples at 517 nm.

### 2.4 Ascorbic acid content

Ascorbic acid was measured according to Klein and Perry (1982) method with minor modifications. First, 5 g of dry leaves were extracted in a methanol solution with a Soxhlet device for 4 h. The resulting extract was passed through filter paper and concentrated with a rotary device at a temperature of 40°C. 50 mg of the resulting extract was extracted with 50 mL of 1% metaphosphoric acid for 45 m. One milliliter of the filtered extract was mixed with 9 mL of the prepared solution, and the absorption number was read at a wavelength of 530 nm.

### 2.5 Total protein and carbohydrates

Lowry's method was used to determine total protein (Lowry et al., 1951). This test is based on the hydrolysis of proteins and the release of amino acids in the protein structure, which creates a colored complex with Fulen's reagent. Finally, the color intensity was measured by a spectrophotometer at a wavelength of 660 nm.

For total carbohydrates, a 0.5 g plant sample was extracted using 5 mL of 80% methanol. The extract was then centrifuged at 6000 rpm for 10 min. Three milliliter of anthrone solution was added to 100 μL of the supernatant, and readings were made at a wavelength of 630 nm (Prasad et al., 1993).

### 2.6 Appearance quality of samples after drying

The results were expressed as Hunter color indices (l*: brightness, a*: green to red symbol, and b*: blue to yellow symbol). The total color difference (Δ*E*) was calculated using the following equation and was used to estimate the color change during drying (Nadian et al., 2015).

(5) $$∆E=\sqrt{{\left(l^{*}-l_{i}^{*}\right)}^{2}+{\left(a^{*}-a_{i}^{*}\right)}^{2}+{\left(b^{*}-b_{i}^{*}\right)}^{2}}$$

The l* index is a symbol of color brightness and varies from 0 for black color to 100 for white color. The range of changes of symbol a* is from negative values for green color to positive values for red color, and symbol b* is from negative values for blue color to positive values for yellow color. The hue angle (in degrees) is defined as 0° for red‐pink, 90° for yellow, 180° for gray‐green, and 270° for blue color (Ebrahimi & Miri Karbasak, 2016).

In addition, color intensity or saturation (Chroma ‐ luminance index) and hue angle were determined using the following equations (Hammond, 2016):

(6) $$\text{Chroma}=\sqrt{\left(a^{*2}+b^{*2}\right)}$$

(7) $$\mathrm{Hue}\;\text{angle}=\tan^{-1}\left(\frac{b^{*}}{a^{*}}\right)$$

Based on the three parameters l*a*b*, the whiteness index (WI) was calculated as follows.

(8) $$\mathrm{WI}=100-\sqrt{{\left(100-L^{*}\right)}^{2}+{a^{*}}^{2}+{b^{*}}^{2}}$$

This index shows the tendency of the samples to be light (white), and the closer its value is to 100, the lighter the samples are.

In this experiment, the browning index was also calculated using Equation 9. The value of *x* in Equation 9 was determined using Equation 10 (Ergüneş & Tarhan, 2006). Equation 11 was also used to calculate the redness index (Pathare et al., 2013).

(9) $$\mathrm{BI}=\frac{\left[100\left(x-0.31\right)\right]}{0.17}$$

(10) $$x=\frac{\left(a^{*}+1.75l^{*}\right)}{\left(5.645l^{*}+a^{*}-3.012b^{*}\right)}$$

(11) $$\mathrm{RI}=\frac{a^{*}}{b^{*}}$$

### 2.7 Statistical analysis

Finally, the experimental data were analyzed using SAS 9.3, and the comparison of means was conducted using the LSD test at 95% confidence intervals. Path analysis was utilized to estimate the direct and indirect effects of traits on the browning index using Path 2 software following the Dewey and Lu method (Dewey & Lu, 1959). The correlation between the browning index and other traits was calculated using SPSS 14.0.

## 3 RESULTS AND DISCUSSION

### 3.1 Photosynthetic pigments

The main effect of the drying method indicated that the content of photosynthetic pigments changed more in the shade compared to other conditions. The shade‐dried samples had the highest content of carotenoids (2.57 mg/g). The lowest amount of carotenoid (1.74 mg/g) was related to samples dried in a vacuum at 55°C. Moreover, based on storage time, as the storage period of the plant sample increased, the amount of carotenoids increased, so that the highest level of carotenoids (2.19 mg/g) was found at the end of the 150‐day storage period (Table 1). The result of the interaction drying method and storage time in Table 2 shows that as the storage time increased, the level of plant pigments decreased. The samples dried in the vacuum oven had a higher content of chlorophyll a (3.40 mg/g) and total (4.78 mg/g) compared to other samples. Chlorophyll b (1.47 mg/g) was higher in the oven‐dried samples, and the shade‐dried samples maintained for 150 days had the lowest content of photosynthetic pigments.

**TABLE 1:** Effects of different treatments on the content of photosynthetic pigments and biochemical compounds of *Eryngium caeruleum*.

Treatments	Levels	Carotenoids (mg/g)	Total flavonoid (mg/g)	Antioxidant activity (μg/g)	Ascorbic acid (μg/g)	Total protein (%)	Total carbohydrates (μg/g)
Drying method	Shade	2.57^a^	46.5^c^	66.66^b^	228.03^c^	18.28^b^	52.74^a^
	Oven 55°C	2.03^b^	50.2^ab^	69.85^a^	314.73^a^	19.32^a^	40.75^b^
	Vacuum oven 55°C	1.74^c^	50.6^a^	70.15^a^	316.90^a^	19.27^a^	42.26^d^
	Microwave 500 W	2.06^b^	49.3^b^	69.68^a^	296.83^b^	19.15^a^	47.84^c^
Storage time (day)	1	2.01^b^	–	68.95^b^	295.51^a^	–	–
	75	2.11^ab^	–	70.77^a^	288.57^ab^	–	–
	150	2.19^a^	–	67.54^c^	283.29^b^	–	–

*Note*: Different letters in each column indicate a significant difference by LSD at the 5% level.

**TABLE 2:** Effect of drying method × storage time on the content of photosynthetic pigments of *Eryngium caeruleum*.

Drying method	Storage time (day)	Chlorophyll a (mg/g)	Chlorophyll b (mg/g)	Total chlorophyll (mg/g)
Shade	1	2.63^b^	0.94^ef^	3.57^b^
	75	2.13^e^	0.86^fg^	2.99^ef^
	150	2.07^e^	0.72^h^	2.79^f^
Oven 55°C	1	3.30^a^	1.47^a^	4.77a
	75	2.37^c^	1.19^c^	3.56^b^
	150	2.27^cde^	0.95^ef^	3.22^cde^
Vacuum oven 55°C	1	3.40^a^	1.38^ab^	4.78^a^
	75	2.34^cd^	1.08^d^	3.42^bc^
	150	2.28^cde^	0.93^ef^	3.22^cde^
Microwave 500 W	1	3.22^a^	1.35^b^	4.57^a^
	75	2.36^cd^	0.98^de^	3.34^bcd^
	150	2.29^cde^	0.82^gh^	3.11^de^

*Note*: Different letters in each column indicate a significant difference by LSD at the 5% level.

During the drying process, the color of plants can change due to the degradation of carotenoids and non‐enzymatic reactions (Onwude et al., 2016). Factors such as long processing times, high temperatures, and seasonal changes can contribute to the degradation of carotenoids. Different drying methods can affect the content of chlorophyll and carotenoids differently (Md Nor, 2013). Shade‐dried samples may experience higher chlorophyll degradation due to the longer drying process, while microwave and oven drying, although faster, can intensify chlorophyll degradation due to the generated heat. Generally, the rapid drying speed, leading to reduced input energy, helps prevent the evaporation of essential substances and aromatic components. Prolonged drying periods at higher temperatures typically result in greater loss of photosynthetic pigments, increased carotenoid levels, and a paler plant sample (Borchani et al., 2011; Mashkani et al., 2018). In a study on coriander, microwave‐dried samples had a higher content of carotenoids compared to oven‐dried samples (Divya et al., 2012). In this study, microwave‐dried samples had a higher content of carotenoids and a lower content of chlorophyll compared to oven‐dried samples and vacuum oven‐dried samples. The degradation of carotenoids not only affects the color of the crop but also its nutritional value and taste. It is important to note that the specific changes in carotenoid content depend on the nature of the plant material (de Ancos et al., 2018).

### 3.2 Phytochemical compounds

**FIGURE 1:**
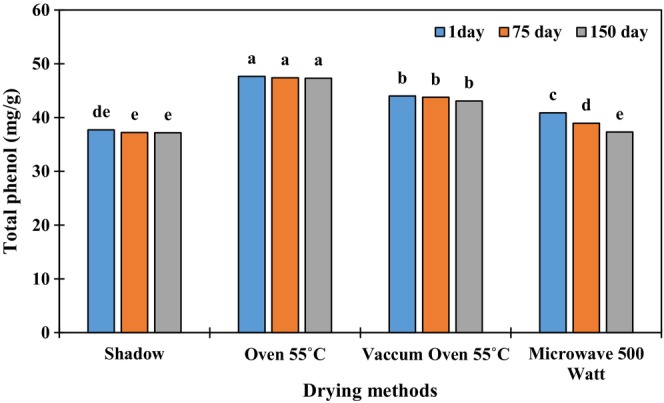
Comparison results of the average interaction effect of different drying methods and storage times on the amount of total phenol in *Eryngium caeruleum*. Different letters in each column indicate a significant difference by LSD at the 5% level.

The level of total flavonoids, as well as the antioxidant activity, in samples dried in a vacuum oven at 55°C (50.6 mg/g), was higher than in the other samples. The lowest content of the phytochemical compounds was observed in shade‐dried samples. The antioxidant activity of *E. caeruleum* significantly got bigger with increasing the storage time up to 75 days (70.7 μg/g), but its lowest was observed on the 150 days (Table 1). Moreover, shade‐dried samples had the lowest content of total phenolics (37.9 mg/g) after 150 days of storage. The highest total phenolics (47.7 mg/g) were found in the other drying methods after 75 days of storage (Figure 1).

In post‐harvest processes, drying methods play an important role in the formation of secondary metabolites, including phenolic acids. (Koca et al., 2007). In the present research, shade drying decreased the content of phenolic compounds, hence the antioxidant activity. High temperatures reduce enzymatic activities, especially the activity of antioxidant enzymes. After drying with a vacuum oven at 55°C and with an oven at 55°C, the content of phenolics and flavonoids and the antioxidant activity were at their maximum levels. However, storing the plant materials for a longer period led to the destruction of these compounds. In the spinach reported, the content of flavonoids decreased during storage (Martínez‐Sánchez et al., 2008). According to recent findings, storage and high drying temperatures, and, as a result, shorter processing times, have an important effect on the content of phenolic compounds (Deng et al., 2018; Zhao et al., 2017). In another study, the highest content of phenolic compounds, rosmarinus acid, and antioxidant properties in the mint family were reported with the natural drying method (Hossain et al., 2010). In the lemongrass plant, shade‐drying compared to sun‐drying and different oven temperatures helped to preserve the phenolic compounds (Mohtashami et al., 2012). Hossain et al. (2010) reported that high‐power microwaves lead to the release of these compounds by changing or destroying the structure of the membranes. In the microwave method, probably due to the high drying speed of the samples and lack of sufficient time for breaking the cell structure and better extraction of phenolic compounds, the content of these compounds was lower than with the oven method. In some cases, the increase in antioxidant capacity during storage can be due to the formation of new compounds with higher antioxidant activities, such as Maillard reaction products, which occur in long‐term storage (Häkkinen et al., 2000).

### 3.3 Biochemical compounds

Vacuum oven‐dried and oven‐dried samples had the highest content of ascorbic acid (316 μg/g) and protein content (19.3%), which was nonsignificant together. The lowest amount of ascorbic acid (228 μg/g) and protein content (19.3%) and highest content of total carbohydrates (52.7 μg/g) were observed in the shade‐dried samples. Moreover, the content of ascorbic acid decreased (from 29.5 to 283.3 μg/g) with increasing storage time (Table 1). Ajayi et al. (1980) found that six medicinal plants experienced a decrease of 52%–81% in ascorbic acid levels over time. The study revealed that vitamin C content rose using the oven method (38.02%) and the vacuum method (38.97%) in comparison to shade‐drying. However, the levels decreased after 75 days (2.35%) and 150 days (4.13%). This variation may stem from differences in plant characteristics (López et al., 2010).

### 3.4 Colorimetric indices

**FIGURE 2:**
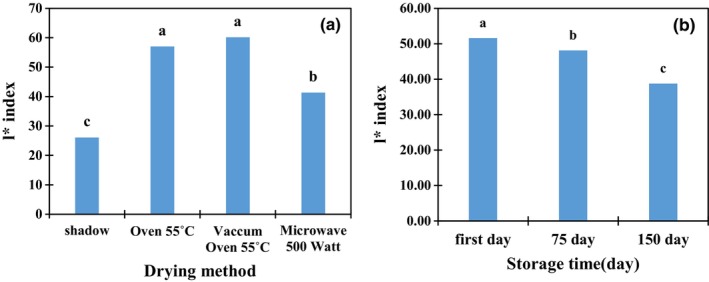
Mean comparison of l* index under the simple effects of drying method (a) and storage time (b). Different letters in each column indicate a significant difference by LSD at the 5% level.

The l* index, which represents brightness, was higher in vacuum oven‐dried samples, followed by oven‐dried samples, compared to other treatments. Over time, the l* index decreased, and the samples tended to turn black (Figure 2).

The interactive effect of the drying method and storage time on the a* and b* indices was also significant. Shade‐dried samples with a storage period of 150 days had a higher a* index (45.7), indicating a somewhat red color. On the other hand, the b* index (blue‐yellow) in shade‐dried samples decreased significantly after 150 days (from 9.67 to −3.0), indicating blueness, while vacuum oven‐dried samples showed a yellow color on the first day (Table 3).

**TABLE 3:** Effect of drying method × storage time on the colorimetric indices l*a*b* of *Eryngium caeruleum*.

Drying method	Storage time (day)	Colorimetric indices		Color characteristics			Color indices		
		a*	b*	∆*E*	SI	Hue	WI	BI	Reddish
Shade	1	17.33^f^	9.67^h^	53.57^de^	20.09^g^	−1.51^f^	27.44^ef^	77.33^de^	−2.42^c^
	75	37.33^cd^	5.33^i^	68.34^b^	37.76^ef^	1.04^c^	16.91^g^	107.67^bc^	2.79^ab^
	150	45.67^a^	−3.00^j^	79.83^a^	45.79^c^	1.10^bc^	9.54^h^	128.52^a^	8.06^a^
Oven 55°C	1	−30.67^h^	40.00^b^	29.89^f^	46.54^bc^	−0.65^e^	38.13^ab^	44.92^f^	−0.76^b^
	75	29.93^e^	37.93^bc^	33.96^f^	48.32^bc^	0.67^d^	37.66^ab^	111.84^abc^	0.79^ab^
	150	42.93^ab^	17.93^fg^	55.71^cd^	50.46^b^	1.17^b^	28.51^de^	127.77^a^	2.40^ab^
Vacuum oven 55°C	1	−32.00^h^	47.11^a^	32.24^f^	44.58^cd^	−0.60^e^	41.96^a^	64.45^e^	−0.68^b^
	75	28.11^e^	35.11^c^	31.45^f^	44.98^c^	0.67^d^	33.78^bcd^	103.82^c^	0.80^ab^
	150	39.78^bc^	20.11^ef^	50.04^de^	57.00^a^	1.43^a^	33.46^bcd^	109.88^abc^	1.98^ab^
Microwave 500 W	1	−21.67^g^	27.33^d^	34.54^f^	35.00^f^	−0.67^e^	35.07^bc^	40.41^f^	−0.80^b^
	75	33.33^de^	22.33^e^	48.48^e^	40.25^de^	0.98^c^	29.63^cde^	94.66^cd^	1.51^ab^
	150	42.00^abc^	15.33^g^	60.74^c^	44.75^cd^	1.22^b^	22.16^fg^	125.75^ab^	1.88^ab^

*Note*: Different letters in each column indicate a significant difference by LSD at the 5% level.

The drying process affects the surface properties of plant materials and thus changes their ability to reflect light and color (Zamani et al., 2023). High temperatures and oxidation during drying cause chemical changes in chlorophyll and carotenoid pigments (Borchani et al., 2011; Fernandes & Rodrigues, 2008). According to the colorimetric results, the different drying methods changed the color of the samples. The highest brightness index (l*) was found in the vacuum oven‐dried samples, followed by those dried in the conventional oven and 500‐W microwave oven. According to the results, as the drying process shortened, the l* increased. With increasing temperature/ drying time, the a* increased/decreased. Finally, samples dried in the vacuum or conventional oven better maintained their greenness. Zielinska and Michalska (2016) reported that drying temperature has a significant effect on the color of medicinal and aromatic plants. Mirmostafaee (2014) also reported that the color characteristics of fresh and dried peppermint plants are influenced by the drying air temperature and the microwave oven, the color of the leaves was preserved to a greater extent than in the sun‐drying method. Researchers showed that oven‐drying at high temperatures led to a decrease in the color quality of thyme leaves, while microwave‐drying in the shortest time resulted in high color quality and a significant increase in the bioactive compounds (Rahimmalek & Goli, 2013). Topuz et al. (2009) observed that freeze drying was the optimal method to preserve color because pigments were not destroyed by heat and oxidation. A higher temperature accelerated the non‐enzymatic browning reactions, and subsequently, yellowness increased (Mounir et al., 2020). The decrease in l* value is due to non‐enzymatic browning reactions and the formation of brown pigments due to the Maillard reaction (Baini & Langrish, 2009).

In samples dried at 55°C, due to the high temperature and the intensity of the Maillard reaction, the brightness (l*) was expected to be lower than in the other samples but increased in all test conditions. The redness of plant samples increased with all drying methods. Its initial values at the beginning of storage were smaller than those at 75 and 150 days. After drying and the storage period, its values were greater than the initial values, indicating that the color changed toward red. The yellowness of plant samples (b*) increased after storage in all test conditions. Yellowness during drying time increased according to the rate of decrease in moisture of the product, which can be related to the degradation of chlorophyll, which increases the carotenoid concentration of the dried samples. Thus, the color of the samples turns yellow (Bahmanpour et al., 2017; Zielinska & Michalska, 2016).

### 3.5 Color properties

The overall color change in the shade‐dried samples after 150 days of storage was found to be higher than in the other samples. The highest chroma index was observed in the samples dried in the vacuum oven at 55°C and kept at storage conditions for 150 days. The highest Hue angle (1.43) was found in the vacuum oven‐dried samples kept at storage conditions for 150 days (Table 3).

The effect of the drying method on brightness was significant (*p* < .05). While the storage time had a significant difference on the browning index (*p* < .05), the interactive effect of the treatments on all three studied indices was significant (*p* < .05). The highest brightness index was found in vacuum oven‐dried samples at the beginning of storage, and the highest browning index was recorded for the shade‐dried samples (128.5) and oven‐dried samples (127.5) after 150 days of storage. Moreover, the highest value of the redness index was observed in the shade‐dried samples after 150 days of storage (Table 3).

Overall color change (Δ*E*) is a combination of color parameters and includes brightness, yellowness, and redness. As previously mentioned, high temperatures and long drying periods are favorable to browning reactions, decreasing the brightness and accelerating color change (Vega‐Gálvez et al., 2012). Increasing Hue showed that *E. caeruleum* became lighter and lost its green color, confirming the destruction of chlorophylls during storage. Since the Maillard reaction depends on the temperature and the intensity of the thermal behavior, drying at a high temperature, compared to a lower temperature, and prolonged drying lead to more color change. Prolonged drying (at low temperatures) also provides sufficient opportunity for the Maillard reaction and the formation of more brown pigments (Nadian et al., 2015). This increase in browning can be attributed to the Maillard reaction and/or an increase in the concentration of carotenoids, both enzymatic and non‐enzymatic (Contreras et al., 2008). Interestingly, the browning decreases at high temperatures (55°C) compared to lower temperatures. This could be due to lower degradation or decomposition of pigments (Ergüneş & Tarhan, 2006). It was also determined that the effect of the “*b*” component on the browning index (BI) is greater than that of “a*” and the effect of “a*” is greater than that of “l*.” Therefore, a higher “b*” value in shade‐dried samples and long‐term storage can counteract the increase in “l*,” resulting in reduced BI.

**FIGURE 3:**
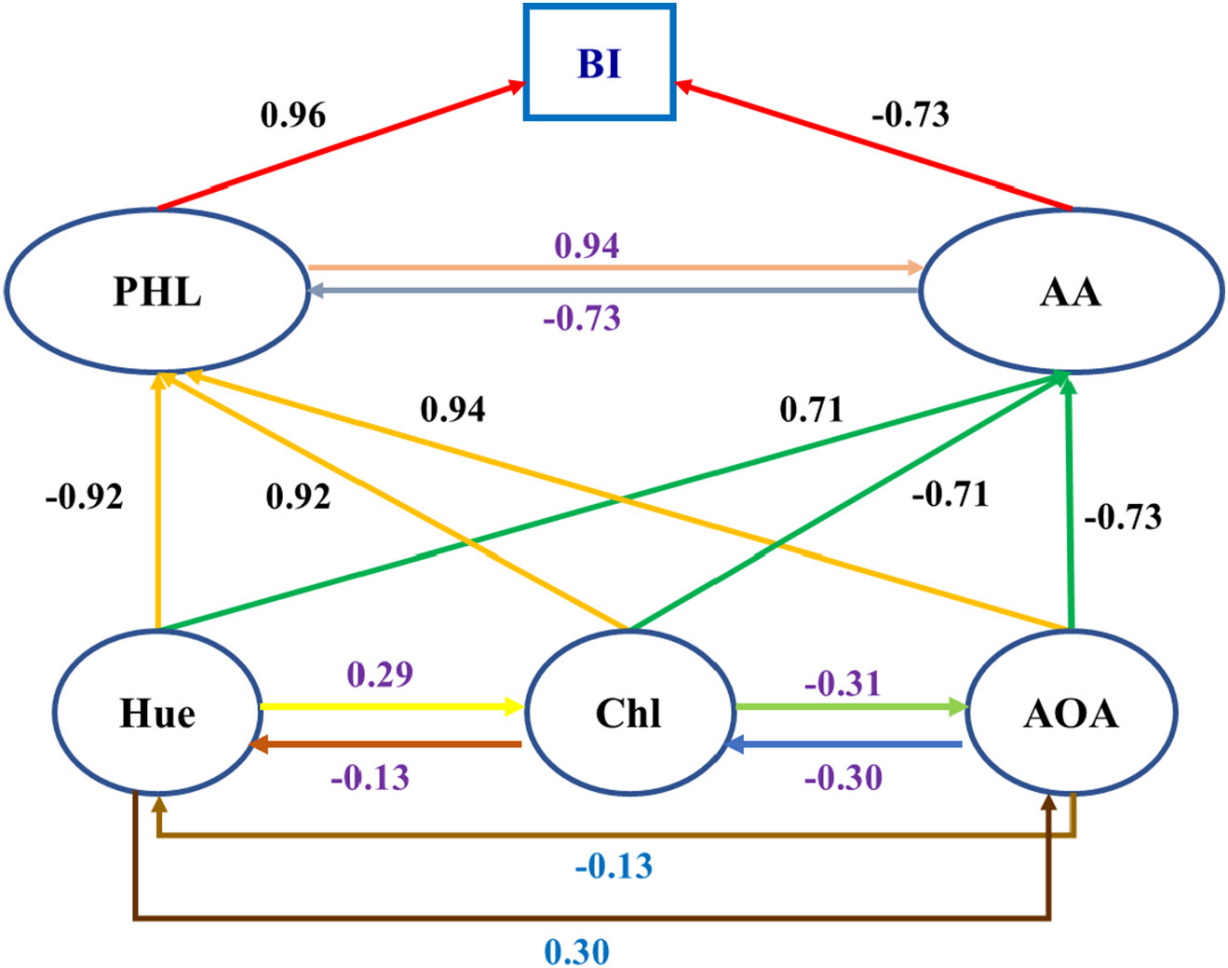
Direct and indirect effects of total phenol (PHL), ascorbic acid (AA), total chlorophyll (Chl), antioxidant activity (AOA), and hue on browning index (BI).

The path analysis in the study provided valuable insights into the factors affecting the browning index of *E. caeruleum* during the drying process. Findings showed significant positive correlations between the browning index and five traits, excluding Hue (Table 4, Figure 3). Total phenol content and ascorbic acid concentration were identified as primary factors directly influencing the browning index. Moreover, indirect effects on the browning index were observed for total chlorophyll, antioxidant activity, and hue, mediated by total phenol and ascorbic acid. While total phenol and ascorbic acid had direct impacts on the browning index, total chlorophyll, antioxidant activity, and hue affected it indirectly through their influence on total phenol and ascorbic acid levels (Bobo et al., 2022; Garcia & Barrett, 2002). The study suggests that increased ascorbic acid levels and decreased total phenol levels may reduce the browning index of *E. caeruleum*, with higher Hue also playing a role in reducing browning. Additionally, optimal levels of total chlorophyll and antioxidant activity were associated with lower browning index values. These results support previous research highlighting the role of ascorbic acid in preventing browning by inhibiting polyphenol oxidase activity (Akbari & Gholami, 2018). Furthermore, the study underscores the potential threshold effect of antioxidants, which, in excess, may disrupt oxidative processes crucial for creating appealing pigments or flavor compounds in food items (Zhang et al., 2023).

**TABLE 4:** Correlation between browning index (BI), total phenol (PHL), ascorbic acid (AA), total chlorophyll (Chl), antioxidant activity (AOA), and hue and path analysis of this variable on browning index (BI).

Variable	BI	Hue	Chl	PHL	AOA	AA	Effect		
							Total	Direct	Indirect
BI	1						–	–	–
Hue	−0.90**	1					−0.90**	0.14	−1.03
Chl	0.87**	−0.95**	1				0.87**	−0.30	1.17
PHL	0.94**	−0.97**	0.97**	1			0.94**	0.95**	−0.01
AOA	0.91**	−0.96**	0.98**	0.99**	1		0.91**	−0.31	1.22
AA	0.93**	−0.97**	0.97**	0.99**	0.99**	1	0.93**	−0.73**	1.66

* and ** are significant at 5% and 1%, respectively.

## 4 CONCLUSIONS

The effect of drying methods and storage duration on *E. caeruleum* has been studied. Decreasing drying time and increasing temperature resulted in a higher content of photosynthetic pigments. Drying at 55°C (in a vacuum oven or regular oven) showed optimal physiological and phytochemical characteristics with the lowest browning index. Longer storage periods reduced brightness and increased browning and redness. Vacuum oven drying at 55°C yielded the best physicochemical results. Among the studied conditions, drying in an oven at 55°C without long‐term storage was found to be the most favorable. Drying and storage processes increased carotenoid pigment content, leading to a higher b* value. Faster drying rates also increased the b* value. Therefore, using high‐speed drying methods and limited storage (around 75 days) can help preserve the color and appearance of *E. caeruleum*. Browning, lightening, redness indices, and appearance quality indexes can be used as a quick and initial step in evaluating the quality of dried leafy products.

## AUTHOR CONTRIBUTIONS

**Soudabeh Nourzad:** Data curation (equal); formal analysis (equal); methodology (equal); resources (equal); software (equal); writing – original draft (equal). **Hassanali Naghdi Badi:** Project administration (equal); supervision (equal); writing – original draft (equal). **Sepideh Kalateh Jari:** Project administration (equal); supervision (equal); writing – original draft (equal); writing – review and editing (equal). **Ali Mehrafarin:** Writing – original draft (equal); writing – review and editing (equal). **Sakineh Saeidi‐Sar:** Writing – review and editing (equal).

## CONFLICT OF INTEREST STATEMENT

The authors declare no conflicts of interest.

## ETHICS STATEMENT

This study does not involve any human or animal testing.

## Data Availability

No separate data are available. The details of any study in this article can be requested by contacting the respective author.
